# Can Tics be Performed Convincingly by an Actor?

**DOI:** 10.1155/2014/893859

**Published:** 2014-05-07

**Authors:** Kirsten R. Müller-Vahl, Laura Riemann, Hermann Krämer, Alexander Münchau

**Affiliations:** ^1^Clinic of Psychiatry, Social Psychiatry and Psychotherapy, Hannover Medical School, Carl-Neuberg-Straße 1, 30625 Hannover, Germany; ^2^German Tourette Self Help Group (Tourette Gesellschaft Deutschland, TGD e.V.), c/o Clinic of Psychiatry, Social Psychiatry and Psychotherapy, Hannover Medical School, Carl-Neuberg-Straße 1, 30625 Hannover, Germany; ^3^Department of Movement Disorders and Neuropsychiatry in Children and Adults, University of Lübeck, Maria-Goeppert-Straße 1, 23562 Lübeck, Germany

## Abstract

*Background*. In the German movie “Vincent will Meer” a healthy actor portrays a man with Tourette's syndrome. *Objective*. The aim of this study was to investigate whether the performance of tics is convincing and whether this judgment depends on whether he/she suffers from tics or not. *Methods*. While the movie was broadcasted in German cinemas, we put an online survey (including 28 questions on different aspects related to the observation, performance, and authenticity of tics) on the web pages of the German self-help group. 276/296 surveys submitted could be used for further analyses. *Results*. 95.7% of all participants felt that the performance of tics was convincing. However, people with tics (*n* = 26) were less convinced compared to those who had never met a person with tics (*n* = 110) (*P* = 0.020). *Conclusions*. Our results further support the hypothesis that tics are not “abnormal” but “physiological” movements that are only misplaced both in time and context.

## 1. Introduction

In spring 2010 the movie “Vincent will Meer” by Ralf Huettner was broadcasted in German cinema. It tells the story about three antiheroes who met while being treated in a mental institution: Marie (portrayed by Karoline Herfurth) suffers from anorexia, Alexander (Johannes Allmayer) from obsessive-compulsive disorder, and Vincent (Florian David Fitz, who does not have tics but mimics them) from Tourette's syndrome (TS). After Marie has stolen the director's car, they embark on a journey to Italy. During this trip initial aversion turns into friendship. However, the director and Vincent's father, a successful politician who has placed Vincent into the institution after his mother's death, follow them. The movie not only comprises funny situations but also is thought-provoking and touching. Finally, the father not only finds his son geographically but also is able and willing to accept him despite the TS and no longer considers him as a loser.

We were interested in whether the performance of tics by a healthy actor is convincing and whether an observer's judgment depends on whether he/she suffers from tics or not.

## 2. Methods

For six weeks (2010-05-10 to 2010-06-18), we put an online survey on the web pages of the German self-help group (http://www.tourette.de, http://www.tourette-gesellschaft.de). Subsequently several German film web pages were linked to these sites. The survey included 28 questions: 8 about the authenticity of the tic performance, 12 related to the fact whether the observation of Vincent's “tics” induced new or—as the case may be—influenced existing tics, and 8 general and personal questions (e.g., how people liked the movie, whether they have tics or know others with tics, gender, and age).

## 3. Results

296 people activated and submitted a survey: 276 surveys could be used for further analyses; 20 had to be excluded due to inconsistencies. 37 (13.4%) people were <18 years old, 110 (39.9%) between 19 and 30 years, 117 (42.4%) between 31 and 60 years, and 3 (1.1%) >60 years (missing data: *n* = 9); 76 (27.5%) were male and 193 (69.9%) were female (missing data: *n* = 7). 26 (9.5%) reported that they suffer from tics/TS, 57 (20.7%) reported that they know other people with tics well, 72 (26.1%) answered that they have met others with tics personally, and 110 (39.9%) answered that they have never met someone with tics (missing data: *n* = 11). Answers of participants with tics/TS were compared to the whole group of people without tics (*n* = 250) (using *χ* ^2^ or Welch two-sample *t*-tests) and to the other subgroups (using univariate analyses of variance and Tukey contrasts).

Among those with tics/TS, there were significantly more males than females (*χ* ^2^ = 23.84; df = 1; *P* = 0.000). Altogether, 92.7% stated that they liked the film very much, and 56.2% stated that they were interested in the theme. Interestingly, people with tics/TS liked the film significantly less compared to those without (*t* = 2.39; df = 27.2; *P* = 0.024). While 92.8% were convinced that the actor Florian David Fitz did not suffer from TS, only 82.2% stated that he has no tics at all (independent of whether people suffered from TS or not). 51.8% believed that Vincent suffered not only from tics but also from other “extra movements.” This opinion was held significantly more often by people without TS compared to those with TS (*t* = 2.41; df = 31.39; *P* = 0.022).

95.7% felt that Florian David Fitz' performance of tics was convincing. However, people with tics were significantly less convinced compared to those who had never met a person with tics (*F* = 3.33; df = 3.260; *P* = 0.020). When comparing people without tics to those with tics, a trend towards a significant difference was detectable (*t* = 1.71; df = 26.12; *P* = 0.098). 37.3% of the moviegoers (with no difference between people with and without TS) believed that Vincent's tics differ from “real” tics because “real” tics occur more suddenly and abruptly (12%) and are more frequent (9.4%). 9.1% and 8.0%, respectively, felt that Vincent's tics were performed with ostentation and were rehearsed. 70.7% of the moviegoers (with no difference between people with and without TS) were convinced that an actor is unable to imitate tics so realistic that these movements are indistinguishable from “real” tics.

Not surprisingly, significantly (*t* = −4.35; df = 24.43; *P* = 0.0002) more people with TS compared to those without answered “yes” to the question “do you recognize yourself when watching Vincent's tics?” 5.8% of all moviegoers reported that they noticed so far unknown tics while watching the movie. 9.4% answered that they felt an urge to imitate Vincent's “extra movements,” and 10.1% had an urge to echo his noises/shouts/coprolalia, with the strongest urge for imitating coprolalia. 6.9% and 7.2%, respectively, reported that they indeed echoed motor and vocal tics (most often coprolalia). Although people with TS (compared to those without) more often felt an urge to tic or indeed echoed tics while watching the movie, differences were not significant. Only for the question “did you indeed echo noises/shouts/coprolalia while watching the movie,” we found a trend towards a significant difference (*t* = −2.03; df = 25.56; *P* = 0.053).

## 4. Discussion

In “Vincent will Meer” for the first time a leading actor portrayed a man with TS in a German movie. The movie was well received in Germany and 881.832 moviegoers saw it (cut-off date 2010-10-10). For his film part of Vincent, Florian David Fitz won both the German Bambi award and the German film award Lola.

Although our results obtained from German moviegoers cannot be considered representative, some interesting conclusions can be drawn. Portraits of mentally ill person and TS seem to be of interest for German moviegoers. It can be speculated that people without TS liked the film better because this group comprises more females than males (while the TS group comprises more males than females since males are affected 3 to 4 times as often as females) [1] and in Germany Florian David Fitz (*1974) is a very popular young actor, especially among young female. The vast majority of the moviegoers (95.7%) stated that the performance of the tics was convincing. In this context it is noteworthy that Florian David Fitz came close into contact with a person with tics because one of his acting instructors suffered from TS. In addition, he carefully prepared his performance by contacting people with TS personally and watching documentaries. However, moviegoers with TS were less convinced by the performance compared to those without (especially to those who had never met a person with tics), probably because people with tics are much better able to put themselves in the position of a man with tics leading to a better distinction between “real” and “performed” tics.

Although people with TS slightly more often stated that they had an urge to tic or to echoed “tics” while watching the movie compared to people without tics, there was no significant group difference. Bearing in mind that the number of people who felt the urge to imitate was small, these findings can probably be explained by the fact that echophenomena commonly occur not only in people with tics but also in those without. In the former group they are typically more obvious, although these people are often not aware of them.

Our result that people with TS stated that they more often echoed vocal than motor tics is in line with this assumption: (1) it is more likely that people become aware of echoes when imitating noises as compared to movements, and (2) since healthy people probably imitate movements more readily than noises, it is likely that the imitation of movements is “nearer” to normal than echoing noises.

People without TS more often believed that Vincent suffers not only from tics but also from other “extra movements” indicating that people with TS are better informed about the spectrum of tics.

Some limitations of this survey have to be addressed: (1) it cannot be excluded that people did not answer truthfully; (2) the number of patients with TS participating in this survey was quite small, but obviously above the expected prevalence rate (9.8% versus 1%) [1]; (3) the group of people with TS comprises more males than females, while there were more females than males among those without TS; (4) people with TS were significantly older than those without. Thus, differences in gender, age, and group size might have influenced our results. Furthermore, results might be biased because people who suffer from severe tics or significant echophenomena did not watch the movie because they do not usually go to the cinema.

In conclusion, this survey suggests that tics can be convincingly imitated by a well-trained healthy actor. However, people with tics are less convinced compared to those without, probably because they are much better able to put themselves in the position of someone with tics. These results further support the hypothesis that tics are not “abnormal” but “physiological” movements that are only misplaced both in time and context [2]. This hypothesis is further supported by the fact that it is more difficult to distinguish “organic tics” from “psychogenic tics” compared to other movement disorders (such as tremor, dystonia, and myoclonus). Since “psychogenic” and “organic” tics share several clinical similarities, additional features have been suggested for differentiation including premonitory sensation, suppressibility, family history, distractibility, and coexistence of other psychogenic movements [3]. It can be speculated that echophenomena are symptoms, “near to normal,” that occur not only in patients with TS but also—albeit to a lesser extent—in healthy people.
